# ﻿A new leafhopper genus and one new species of the genus *Edwardsiana* of Typhlocybini (Hemiptera, Cicadellidae, Typhlocybinae) from China

**DOI:** 10.3897/zookeys.1254.164010

**Published:** 2025-10-01

**Authors:** Bin Yan, Mick D. Webb, Zi-Yue Deng, Xiao-Fei Yu, Zi-Zhong Li, Mao-Fa Yang

**Affiliations:** 1 Guizhou Key Laboratory of Plateau Wetland Conservation and Restoration, Guizhou Normal University, Guiyang, 550025, China; 2 Department of Life Sciences, Natural History Museum (Retired), Cromwell Road, London, SW7 5BD, UK; 3 Institute of Entomology, Guizhou University, Guiyang, 550025, China; 4 College of Tobacco Science, Guizhou University, Guiyang, 550025, China

**Keywords:** Auchenorrhyncha, classification, identification key, microleafhopper, morphology, new combination, new placement, new taxon, taxonomy

## Abstract

The mukariine species *Mohunia biguttata* (Wang & Li, 2003) had been transferred to the subfamily Typhlocybinae, but its tribal placement and generic status remained uncertain. In this study, a new genus, *Ommatocyba* Yan, Yang & Webb, **gen. nov.** is erected for *Mohunia biguttata* as *Ommatocyba biguttata* (Wang & Li), **comb. nov.**, and placed in the tribe Typhlocybini, **new placement**, based on wing venation. In addition, a new species of *Edwardsiana* Zachvatkin, *Edwardsiana wanglangensis* Yan, Yang & Webb, **sp. nov.** (Typhlocybini) from Sichuan, China, is described and illustrated, and a key is provided for its separation.

## ﻿Introduction

The Chinese leafhopper species, *Mohunia biguttata* Wang & Li, 2003 was transferred from the subfamily Deltocephalinae (Mukariini) to the subfamily Typhlocybinae by Chen et al. (2007), but without placement to genus; no further mention of the species has been made in the Mukariini literature (Chen et al. 2008; Viraktamath and Webb 2019; Meshram 2021). Based on additional collected material, we here confirm the placement of the species in Typhlocybinae and describe a new genus for it in the tribe Typhlocybini (new placement). In addition, the opportunity is taken to describe a new Typhlocybini species from China in the genus *Edwardsiana* Zachvatkin, and a key is provided for the separation of the species of this genus from China.

## ﻿Material and methods

The length of the body reported in the descriptions includes the forewings at rest. Morphological terminology follows Dworakowska (1993) and Dietrich (2013). Male specimens were dissected under a LEICA S9i microscope and then transferred to glycerine for further observation. Male genitalia were drawn using a LEICA DM750 compound microscope, and drawings were enhanced using Adobe Illustrator CC 2018. Photographs of the habitus and male pygofer were taken with a KEYENCE VHX-6000 digital camera and a NIKON ECLIPSE Ni-E compound microscope, respectively. All specimens studied are deposited in the Institute of Entomology, Guizhou University, Guiyang, China (**GUGC**).

## ﻿Taxonomic results

**Typhlocybini Kirschbaum, 1868**

### ﻿Genus Ommatocyba

Taxon classificationAnimaliaHemipteraCicadellidae

Yan, Yang & Webb, gen. nov., new placement

60A77A6A-B95C-51F8-A7DE-0A40B3DF313A

https://zoobank.org/B41B06E6-F931-47C7-8822-B061E624ADE1

Figs 1, 2

#### Type species.

*Mohunia biguttata* Wang & Li, 2003.

#### Description.

**Figure 1.**
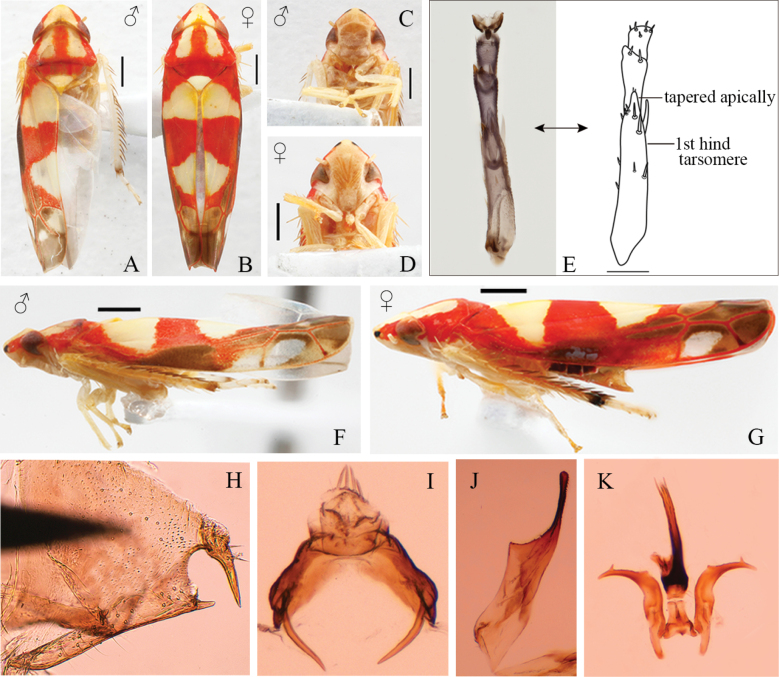
External features and male genitalia of *Ommatocyba biguttata* (Wang & Li, 2003). A, B. Habitus, dorsal view C, D. Face, ventral view; E. Hind tarsomeres F, G. Habitus, lateral view; H. Pygofer side, lateral view; I. Anal tube, caudal view; J. Subgenital plate, ventral view; K. Aedeagus, style and connective, ventral view. Scale bars, except E: 0.5 mm; scale bars of E: 0.1 mm.

**Figure 2.**
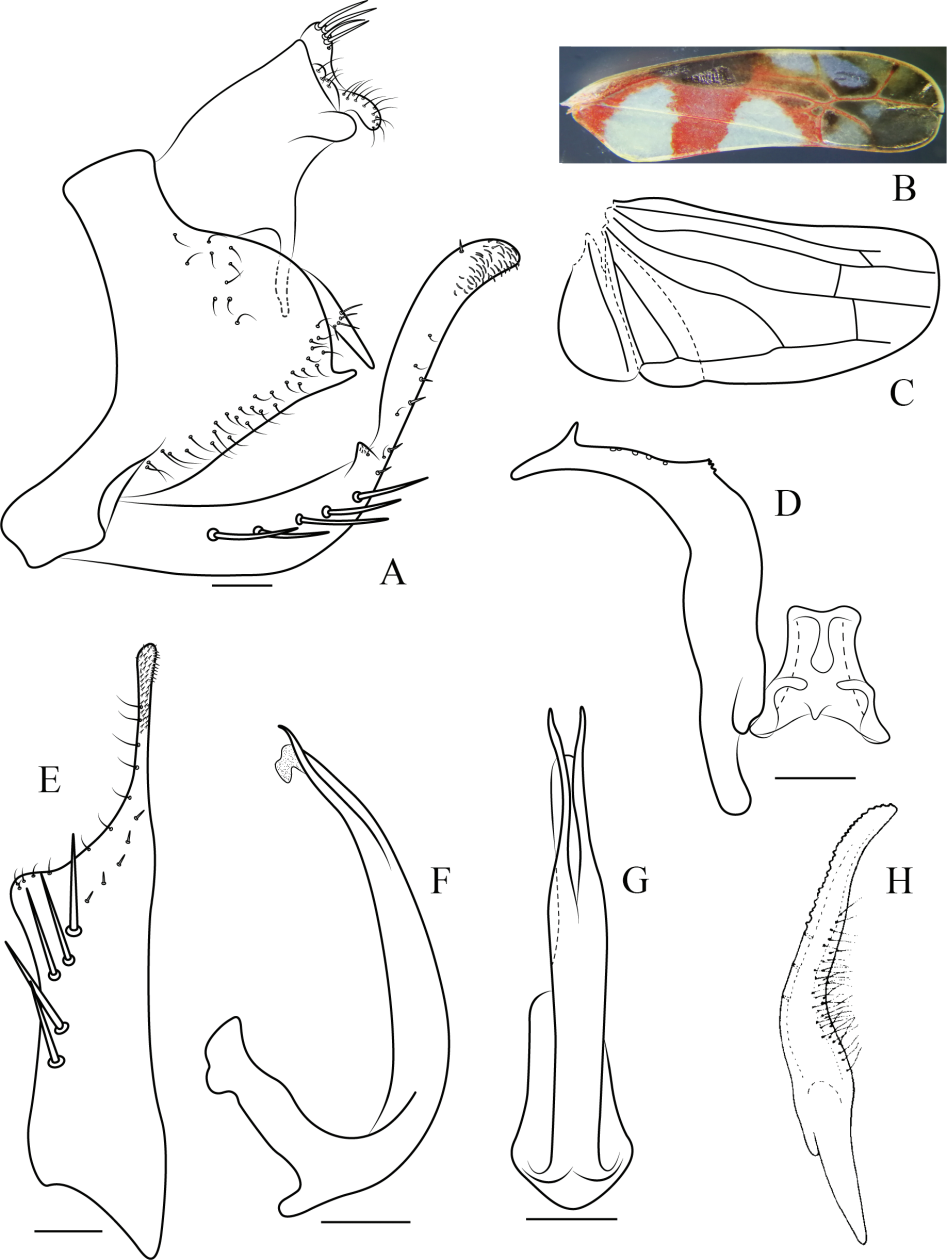
Wings and male genitalia of *Ommatocyba biguttata* (Wang & Li, 2003). A. Genital capsule, lateral view; B. Forewing; C. Hind wing; D. Style and connective, ventral view; E. Subgenital plate, ventral view; F. Aedeagus, lateral view; G. Aedeagus, ventral view; H. Style of *Bolanusoides heros* Distant, 1918 (after Distant, 1918 and Dworakowska, 1988). Scale bars: 0.1 mm.

Body relatively robust, lightly colored with distinct markings. Head compressed, narrow in lateral view, crown produced medially, distinctly shorter than interocular width and slightly narrower than pronotum (Fig. 1A, B). Face slightly inflated, frontoclypeus and anteclypeus in male very broad with lorum and gena very narrow (Fig. 1C, D). Third apical cell of forewing petiolate basally (Fig. 2B). Hind wing veins RP and MA separate throughout length, connected by cross vein distally; submarginal vein not exceeding CuA vein (Fig. 2C).

Male pygofer triangular-shaped in lateral view; dorsal bridge short; lobe with appendages on caudal part, the more dorsal appendage with setae; several microsetae along lower margin of lobe (Figs 1H, 2A). Anal tube with ventral appendage subbasally on each side (Figs 1I, 2A). Subgenital plate with caudal 2/5 narrowed abruptly to inner margin; with uniseriate row of macrosetae on broader basal part followed by a few spine-like setae; inner margin distally with short fine setae (Figs 1J, 2E). Style with outer basal arm short, distal part beyond basal arms long and robust basally, distal third strongly curved laterally and tapered to foot-like apex with subapical serrated lobe on inner margin (Figs 1K, 2D). Connective lamellate, central rib weak (Figs 1K, 2D). Aedeagus with shaft elongate, tapered to apex and weakly curved dorsally in lateral view, with pair of subapical processes on ventral surface, gonopore apical on ventral surface, basal apodeme moderately well developed, compressed antero-posteriorly (Figs 1K, 2F, G).

#### Diagnosis.

The new genus resembles *Bolanusoides* Distant in its compressed head (Fig. 1F, G) and petiolate third (outer) apical forewing cell (Fig. 2B) but can be distinguished from the latter by the following features: hind wing with RP and MA separate throughout length and connected by cross vein (Fig. 2C) rather than fused distally in *Bolanusoides*; postclypeus and anteclypeus distinctly dilated in male (Fig. 1C); unique shape of the subgenital plate (Figs 1J, 2E); style foot-like apically rather than tapered to apex in *Bolanusoides*, with subapical lobe on inner margin, and without fine setae rather than many fine setae on outer margin at midlength in *Bolanusoides* (compare Fig. 2D, H); the subapical and medial regions of the aedeagal shaft are parallel-sided rather than inflated in *Bolanusoides*.

#### Remarks.

Based on its flattened appearance, *Ommatocyba biguttata* (the type species of *Ommatocyba*) was originally placed in the genus *Mohunia* Distant (Deltocephalinae: Mukariini). However, based on the location of ocelli, forewing and hindwing venation, apically acute hind tarsomere I (Fig. 1E), and male genitalia, Chen et al. (2007) transferred it to Typhlocybinae (unplaced to genus). More evidence of this subfamily placement is provided here by the shape of the style, while its petiolate 3^rd^ apical cell of the forewing and hind wing lacking a submarginal vein places the genus in the tribe Typhlocybini. Within this tribe, it is further placed in the *Eupteryx* genus complex based on the hind wing with three cross veins. It forms a new genus based on its flattened head, sexually dimorphic face and unique shape of the subgenital plate (see also Diagnosis).

With the addition of *Ommatocyba*, the tribe Typhlocybini comprises 31 genera from China. A key to these genera is in preparation by the first author, the only previous key being in Zhang (1990: 144) in Chinese, comprising nine genera.

#### Etymology.

The generic name is the concatenation of “*ommato*” (from the Greek noun ὄμμα, -ατος = eye) + “cyba” (from the Greek noun κύβη = head, as in *Typhlocyba*), indicating the presence of ocelli in this typhlocybine genus.

#### Distribution.

China (Guangxi, Hainan, Yunnan).

### ﻿ Ommatocyba biguttata

Taxon classificationAnimaliaHemipteraCicadellidae

(Wang & Li, 2003) comb. nov.

CB47C9EE-CEA2-5DA7-8FC0-F94AE32DC0F1

Figs 1, 2

Mohunia biguttata Wang & Li, 2003: 186, figs 1–7.

#### Material examined.

• 2♂♂ (abdomen detached and lost), China, Yunnan Province, Longling County, 26 July 2002, coll. Maofa Yang; • 2♂♂, China, Guangxi Province, Damingshan Mountain, 14–17 May 2011, coll. Xiaofei Yu and Rong Huang; • 2♂♂, China, Guangxi Province, Huaping National Nature Reserve, alt. 730 m, 5 May 2017, coll. Bin Yan; • 1♀ (abdomen detached and lost), China, Hainan Province, Bawangling National Nature Reserve, alt. 1055 m, 28 April 2017, coll. Bin Yan.

#### Description.

Length of male 4.0–4.3 mm; female 4.3 mm. Crown yellow with a black spot each side of midline anteriorly and reddish band between spot and eye (Fig. 1A, B); eyes red-brownish (Fig. 1A–D); face yellowish to brownish (Fig. 1C, D); thorax reddish marked with yellow as a longitudinal patch on pronotum each side of midline, at apex of scutellum and a transverse patch subbasally across forewing and at apex of clavus (Fig. 1A, B); distal part of forewing infuscate with reddish veins (Figs 1F, G, 2B).

***Male genitalia*.** Pygofer triangular-shaped in lateral view; dorsal bridge short; lobe with rows of microsetae on caudal ventral margin; with two caudal appendages, the more dorsal appendage longer, abruptly curved ventrad, with few setae on medial area (Figs 1H, 2A). Anal tube robust and dilated with elongate ventral processes, processes slightly curved inward in posterior view (Figs 1I, 2A). Style with subapical tooth; distally without microsetae on outer margin, few sensorial pits at inner margin, with serrated lobe on inner margin two-thirds distance from base to apex (Figs 1K, 2D). Connective V-shaped, lateral ledges developed, central rib not exceeding central lobe (Figs 1K, 2D). Aedeagus with dorsal apodeme moderately long; preatrium present, short; shaft slightly curved with apex membranous, subapical processes slender, directed caudally, extending slightly beyond shaft apex, slightly sinuate (Figs 1K, 2F, G).

#### Distribution.

China (Yunnan, Hainan, Guangxi).

### ﻿Genus Edwardsiana

Taxon classificationAnimaliaHemipteraCicadellidae

Zachvatkin, 1929

B31DFE11-E007-5ED0-B2EE-65B85CCAC2ED

Edwardsiana Zachvatkin, 1929: 439.

#### Type species.

*Cicada rosae* Linnaeus, 1758.

### ﻿ Edwardsiana wanglangensis

Taxon classificationAnimaliaHemipteraCicadellidae

Yan, Yang & Webb sp. nov.

2B3734CF-9A1C-5B1F-97CA-F2170EBBF6E4

https://zoobank.org/B08D64E0-9351-498D-AC73-5FA12B3F5D7C

Figs 3, 4

#### Material examined.

***Holotype***, ♂, China • Sichuan Province, Pingwu County, Wanglang National Nature Reserve, 24 July 2016, coll. Bin Yan. ***Paratypes***, • 1♂, 7♀♀, same data as holotype.

#### Description.

**Figure 3.**
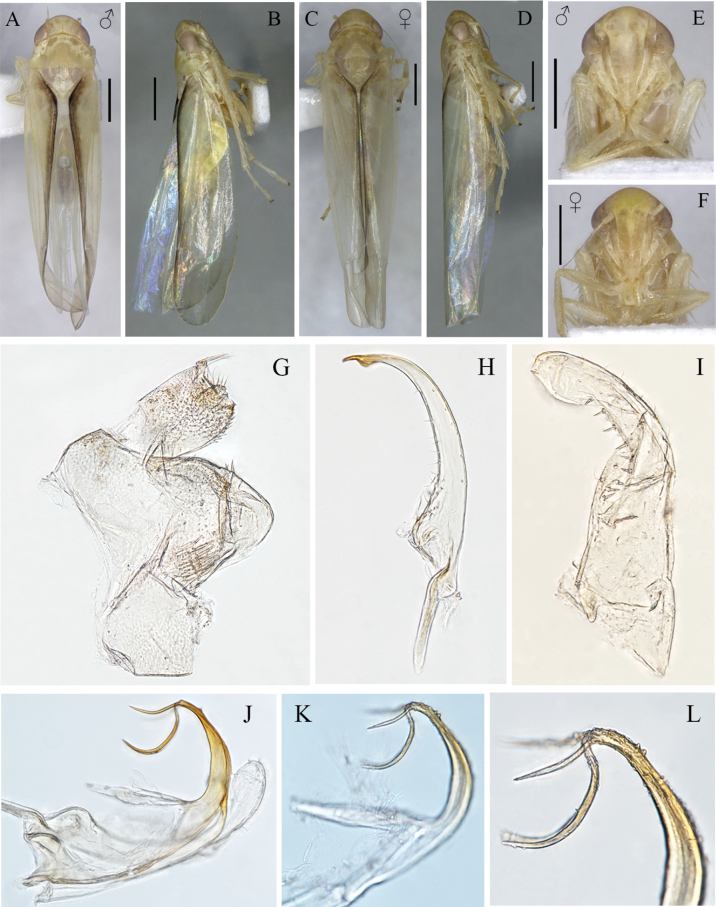
External features and male genitalia of *Edwardsiana wanglangensis* Yan, Yang & Webb, sp. nov. A, B. Habitus of male, dorsal and lateral view, respectively C, D. Habitus of female, dorsal and lateral view, respectively; E. Face of male; F. Face of female; G. Pygofer side, anal tube lateral view; H. Style, ventral view; I. Subgenital plate, ventral view J–L. Aedeagus, lateral view. Scale bars: 0.5 mm.

Length of male 3.6–3.8 mm; female 3.8–4.0 mm. Dorsum pale yellowish (Fig. 3A–D); eyes brownish-grey; face yellowish (Fig. 3E, F); margin of clavus in forewing blackish brown (Fig. 3A, C).

**Figure 4.**
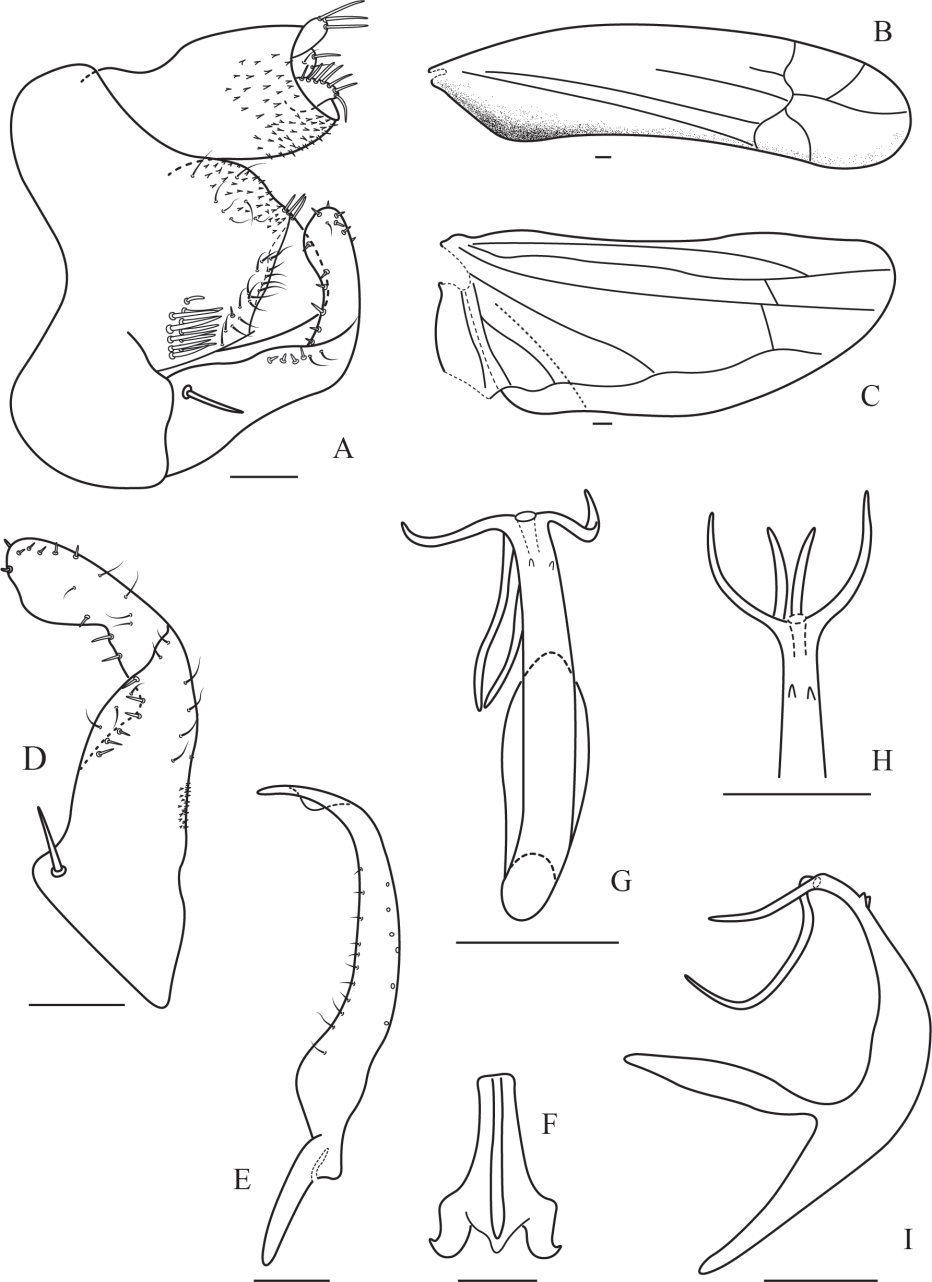
Wings and male genitalia of *Edwardsiana wanglangensis* Yan, Yang & Webb, sp. nov. A. Genital capsule, lateral view; B. Forewing; C. Hind wing; D. Subgenital plate, ventral view; E. Style, ventral view; F. Connective, ventral view; G. Aedeagus, ventral view; H. Aedeagus, apical view I. Aedeagus, lateral view. Scale bars: 0.1 mm.

Crown rounded anteriorly; coronal suture distinct and extending to middle of crown; width of head slightly less than greatest width of pronotum (Fig. 3A, C); forewing with second apical cell largest and third apical cell subtriangular and petiolate (Fig. 4B); hind wing gradually narrowed apically (Fig. 4C).

***Male genitalia*.** Pygofer with dorsal bridge short; pygofer lobe triangular-shaped, without appendage, with a variety of setae: basal region with a group of macrosetae, two rows of microsetae across lobe subapically and several fine setae and macrosetae at posterodorsal region (Figs 3G, 4A). Subgenital plate gradually narrowed towards apex, with distal third abruptly curved dorsad; with one basal macrosetae, row of peg-like setae on lateral margin extended to apex, with several scattered fine microsetae (Figs 3I, 4D). Style tapered distally to acute apex with subapical dilation, with row of microsetae on lateral margin (Figs 3H, 4E). Connective elongate, Y-shaped, central ridge well developed (Fig. 4F). Aedeagus with both preatrium and dorsoatrium well developed, shaft slightly curved dorsally and tapered to narrow apex, with two pairs of elongate processes at apex and two small subapical tooth-like processes on ventral surface (Figs 3J–L, 4I); dorsal margin of longer processes in lateral view with or without a tiny tooth (Figs 3L, 4I), gonopore apical (Fig. 4I).

#### Diagnosis.

This new species is similar to *Edwardsiana ishidai* (Matsumura, 1932) and *E. rosae* (Linnaeus, 1758), but it can be distinguished by the dark brown inner margin of the forewing and aedeagal processes not bifurcate.

#### Etymology.

The species name is derived from the place of collection, Wanglang National Nature Reserve.

#### Distribution.

China (Sichuan).

### ﻿Key to species (males) of the genus *Edwardsiana* from China

**Table:** 

1	Inner margin of forewing dark brown (Figs 3A, 4B); appendages of aedeagus situated on dorsal side of shaft not bifurcated (Fig. 4G)	***Edwardsiana wanglangensis* sp. nov.**
–	Inner margin of forewing yellowish; aedeagal appendages situated on dorsal side of shaft, bifurcate	**2**
2	Aedeagal appendages bifurcate at apex	***Edwardsiana rosae***
–	Aedeagal appendages bifurcate at mid-length	***Edwardsiana ishidai***

## Supplementary Material

XML Treatment for Ommatocyba

XML Treatment for Ommatocyba biguttata

XML Treatment for Edwardsiana

XML Treatment for Edwardsiana wanglangensis

## ﻿Additional information

### Use of AI

No use of AI was reported.

### Author ORCIDs

Bin Yan https://orcid.org/0000-0002-5290-4576

Mick D. Webb https://orcid.org/0000-0002-1312-6142

Zi-Yue Deng https://orcid.org/0009-0001-8114-617X

Xiao-Fei Yu https://orcid.org/0000-0002-1362-9524

Zi-Zhong Li https://orcid.org/0000-0003-1609-9992

Mao-Fa Yang https://orcid.org/0000-0002-5523-6825

### Data availability

All of the data that support the findings of this study are available in the main text.
